# *Chenopodium album* L. and *Sisymbrium officinale* (L.) Scop.: Phytochemical Content and In Vitro Antioxidant and Anti-Inflammatory Potential

**DOI:** 10.3390/plants8110505

**Published:** 2019-11-15

**Authors:** Valentina Amodeo, Mariangela Marrelli, Veronica Pontieri, Roberta Cassano, Sonia Trombino, Filomena Conforti, Giancarlo Statti

**Affiliations:** Department of Pharmacy, Health and Nutritional Sciences, University of Calabria, 87036 Rende (CS), Italy; valentina.amodeo@unical.it (V.A.); mariangela.marrelli@unical.it (M.M.); veronicapontieri@libero.it (V.P.); roberta.cassano@unical.it (R.C.); sonia.trombino@unical.it (S.T.); filomena.conforti@unical.it (F.C.)

**Keywords:** anti-arthritic, anti-denaturation property, antioxidant, bovine serum albumin, *Chenopodium*, nitric oxide, *Sisymbrium*

## Abstract

Spontaneous edible plants have an old history of use in popular traditions all around the world, and the rediscovery of these species could also be useful for the search of new drugs. *Chenopodium album* L. (Amaranthaceae) and *Sisymbrium officinale* (L.) Scop. (Brassicaceae) are two annual plants traditionally used both as food and herbal remedies against inflammatory disorders. In this work, the potential anti-inflammatory and anti-arthritic activities of these plant species have been investigated, together with their antioxidant potential. The phytochemical composition was assessed as well by means of gas chromatography coupled to mass spectrometry (GC-MS) and high performance thin layer chromatography (HPTLC). The antioxidant properties were assessed using the DPPH and β-carotene bleaching test. The ability of extracts to protect against lipid peroxidation was also examined in rat-liver microsomal membranes. All the samples showed a preservation of antioxidant activity up to 60 min. A significant inhibitory activity on the production of the pro-inflammatory mediator nitric oxide was induced in lipopolysaccharide (LPS)-stimulated RAW 264.7 cells by the dichloromethane fraction of *C. album* extract, with an IC_50_ value equal to 81.7 ± 0.9 μg/mL. The same sample showed also a concentration-dependent anti-denaturation effect on heat-treated bovine serum albumin (IC_50_ = 975.6 ± 5.5 μg/mL), even if the best in vitro anti-arthritic activity was observed for the dichloromethane fraction of *S. officinale* extract, with an IC_50_ value of 680.9 ± 13.2 μg/mL.

## 1. Introduction

*Chenopodium album* L. (Amaranthaceae), commonly known as pigweed, is an annual herb growing widely in open habitats such as roadsides and riverbanks [1]. This plant was used in folk medicine as antihelmintic, laxative, as a blood purifier, and it was also used for the treatment of hepatic disorders, intestinal ulcers, and burns [2]. Beside these traditional uses, this species is a known antirheumatic remedy in the traditional medicine of Lebanon. The decoction of its aerial parts mixed with alcohol was utilized against rheumatism and arthritis [3]. *C. album* is common in Italy, where it is also known as fat hen, and traditionally consumed boiled or used in salads, soups, and stews [4].

*Sisymbrium officinale* (L.) Scop. (synonym *Erysimum officinale* L., Brassicaceae) is an annual plant present in Europe, Asia, and northern Africa. It is commonly called hedge mustard, but is also known as “singer’s plant”, because of its traditional use in vocal tract diseases: Flowers and leaves were used for preparing decoctions or tinctures for the treatment of sore throats, coughs, hoarseness, laryngitis, and pharyngitis [5,6]. *S. officinale* is rich in sulfated compounds (particularly glucosinolates, isothiocyanates and sulfated lactones), to which its beneficial properties have been related. However, despite its traditional use, this plant has not yet been deeply investigated [7]. Moreover, shoots and leaves of the wild plant have been traditionally used also as food, in salads [8,9].

Wild edible plants have always been important in the popular traditions of many Mediterranean countries, and ethno-directed research is very useful in the discovery of new drug and food resources [10]. Ethnobotany is a discipline that focuses on the relationships between humans and plants, and it is based on the use of methods from both social and natural sciences. Bioprospecting for new drugs of plant origin, a more powerful tool than random assays for the finding of new active compounds, has classically been based on ethnobotanical information [11,12].

The anti-rheumatic potential of *C. album* was already investigated by Arora and coworkers, who tested the acetone extract of aerial parts on Complete Freund’s adjuvant induced rheumatoid arthritis in rats. The authors reported that the extract was able to induce a significant reduction in rat paw edema (80.13%) after 21 days of treatment at the dose of 200 mg/kg per os, and they also proved that the antirheumatic activity was linked to the inhibition of NF kappa B (NFκB) protein [13].

Politi and colleagues tested the anti-inflammatory activity of *S. officinale* dichloromethane and methanol extracts in the murine Croton oil-induced ear edema model, but just a modest effect was observed at the highest concentrations [14].

Here, in our efforts to investigate the potential health benefits of wild edible plants from Southern Italy [15,16,17,18], we wanted to deeply investigate *C. album* and *S. officinale* biological properties. Together with the antioxidant activity, the potential anti-inflammatory and anti-arthritic activities of these plant species have been investigated. Methanolic extracts and their fractions were tested for their ability to inhibit the lipopolysaccharide (LPS)-induced production of nitric oxide (NO) in the murine macrophage RAW 264.7 cell line, and their capacity to protect bovine serum albumin from heat denaturation. The phytochemical profile has been elucidated as well by means of gas chromatography-mass spectrometry (GC-MS) and high performance thin layer chromatography (HPTLC).

To the best of our knowledge, this is the first report concerning the in vitro anti-denaturation effects on bovine serum albumin for these species.

## 2. Results and Discussion

### 2.1. Phytochemical Profile

The aerial parts of wild *C. album* and *S. officinale* from Southern Calabria (Italy) were extracted with methanol through maceration. Obtained yields were 23.2% and 10.6% for the two raw extracts, respectively (Table 1). A fraction of each crude extract was then successively extracted with solvents with different polarity, *n*-hexane (yield 0.9% for both plant species, referred to dry plant material), dichloromethane (1.6% and 2.2, for *C. album* and *S. officinale*, respectively), and ethyl acetate (0.3%). Remaining aqueous fractions (20.4% and 7.2%) were investigated as well.

The phytochemical content of the apolar fractions was assessed by means of GC-MS. The *n*-hexane samples were particularly rich in fatty acids, being palmitic acid the most abundant one (15.7% and 10.0% of total peak areas in total ion current (TIC) for *C. album* and *S. officinale*, respectively, Table 2).

Stearic acid (1.3% and 1.0%) and arachidic acid (0.8% and 1.0%) were detected to a lesser extent. The other fatty acids were only found at percentages < 1.0%. Two terpenes were also identified in *C. album n*-hexane fraction: The diterpene neophytadiene (0.7%) and the monoterpene dihydroactinidiolide (0.9%) also identified in *S. officinale* sample (0.4%). Moreover, the presence of β-sitosterol was also recognized in *C. album*.

Table 3 reports the chemical composition of the two dichloromethane fractions. The monoterpene lactone loliolide was the most abundant compound in *C. album* (1.7%), followed by methylethylmaleimide (1.4%). This last compound was also identified in *S. officinale*, even if at a lesser extent (0.3%). The phenylpropanoid coniferyl alcohol (1.2%) and the hydroxycinnamic acid ferulic acid (0.7%) were instead the most abundant compounds in *S. officinale*.

**Figure 1:**
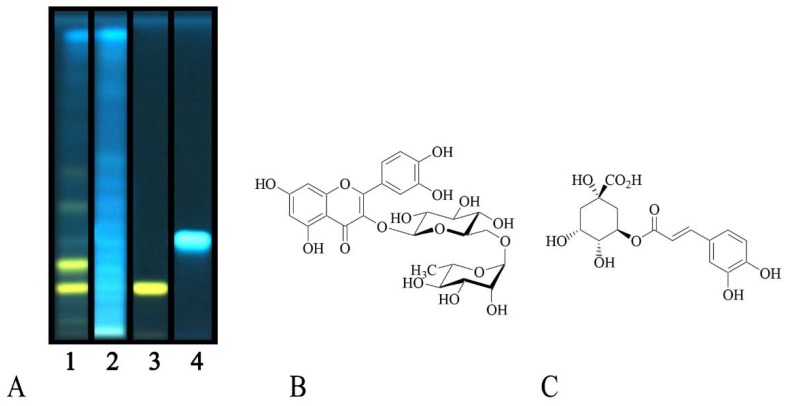
(**A**) High performance thin-layer chromatography (HPTLC) analysis of the ethyl acetate fractions of investigated plants. Mobile phase: Ethyl acetate/dichloromethane/acetic acid/formic acid/water (100:25:10:10:11, v/v/v/v/v). Visualization—366 nm, derivatization—Natural Product Reagent (NPR). Tracks: 1, *C. album* L.; 2, *S. officinale* (L.) Scop.; 3, rutin; 4, chlorogenic acid. (**B**) Rutin. (**C**) Chlorogenic acid.

Total phenolic and total flavonoid contents of *C. album* and *S. officinale* raw extracts were also assessed. The amounts were expressed as chlorogenic acid and quercetin equivalents per g of dry material, respectively. *C. album* showed the highest amounts, with 12.8 ± 1.6 mg/g of phenolic compounds and 0.77 ± 0.01 mg/g of flavonoids, while values of 8.1 ± 0.1 and 0.50 ± 0.01 were observed for *S. officinale* (Table 1), respectively. With the aim to identify the most interesting phenolic compounds, the polar fractions of both plant extracts were then investigated by means of HPTLC, chosen as a practical solution to characterize the complex mixtures of substances present in natural products [19]. Analyses allowed to tentatively identify the presence of rutin and chlorogenic acid (Figure 1). 

**Figure 2:**
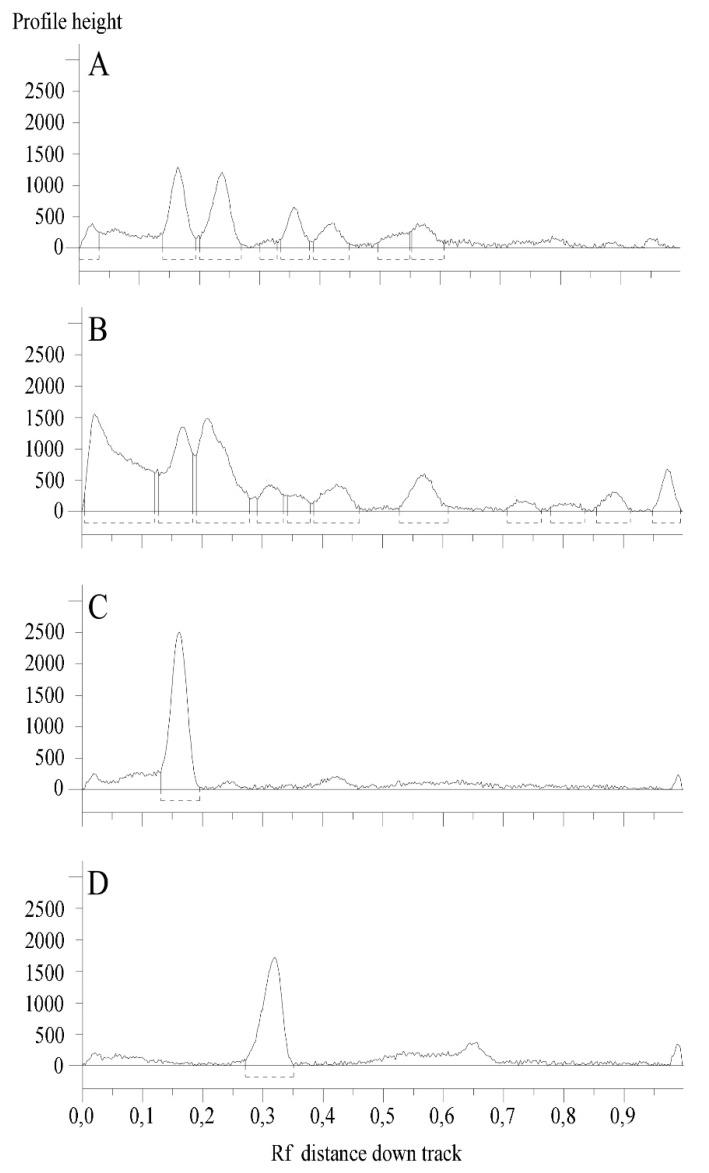
HPTLC chromatograms of analyzed samples and standards. (**A**) *C. album* L.; (**B**) *S. officinale* (L.) Scop.; (**C**) rutin (Rf = 0.16).; (**D**) chlorogenic acid (Rf = 0.32).

The flavonoid glycoside rutin was detected in the EtOAc fraction of *C. album*, as indicated by the typical yellow spot after post chromatographic exposure to NP reagent. Chlorogenic acid was instead identified in the ethyl acetate fractions of both plant species, and it is recognizable as a blue spot in Figure 1. Figure 2 reports the chromatographic profiles of investigated samples and reference standards.

### 2.2. Antioxidant Activity 

The in vitro antioxidant capacities of *C. album* and *S. officinale* extracts and fractions were first evaluated by means of the DPPH and β-carotene bleaching methods. Almost all the samples demonstrated radical scavenging potency. The relation between concentration and percentage inhibition was explained by non-linear regression models. A strong association between concentration and percentage inhibition was observed. As regards the two raw extracts, *S. officinale* (IC_50_ = 143.00 ± 2.61 μg/mL, Table 4) exerted a better radical scavenging potency than *C. album* (IC_50_ = 172.70 ± 2.18 μg/mL, *P* < 0.05, Bonferroni post-hoc test). The best activity was demonstrated by the ethyl acetate fraction of *S. officinale*, with an IC_50_ value of 60.11 ± 1.79 μg/mL. 

This fraction and the EtOAc fraction of *S. officinale* showed an interesting antioxidant activity also in the second test, the β-carotene bleaching method, with IC_50_ values equal to 12.07 ± 0.04. and 12.62 ± 0.75 μg/mL after 30 min of incubation, respectively. The best results were obtained for *S. officinale* raw extract, with an IC_50_ value of 2.61 ± 0.06 μg/mL. This result is particularly interesting if compared with the positive control, propyl gallate. 

**Figure 3:**
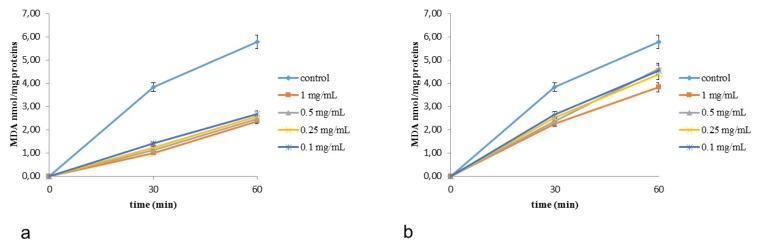
Effects of *Chenopodium album* L. (**a**) and *Sisymbrium officinale* (L.) Scop. extract (**b**) on malondialdehyde (MDA) production induced by tert-butyl hydroperoxide (tert-BOOH) in rat liver microsomal membranes. Results represent the mean ± SEM of four separate experiments. Overall *P* < 0.01.

The capacity of *C. album* and *S. officinale* extracts to protect against lipid peroxidation, induced by tert-butyl hydroperoxide (tert-BOOH), was evaluated in rat-liver microsomes for one hour [20,21,22]. The antioxidant efficiency of extracts were time-dependent and evaluated as MDA production (in nmol mg^−1^ protein, Figure 3). The obtained results indicated that all extracts are effective antioxidants against tert-BOOH-induced lipid peroxidation, showing a preservation of their efficacy up to one hour.

A good antioxidant potential was already reported for *C. album* by Pandey [23], who investigated the biological properties of the petroleum ether, methanol, and aqueous extracts by means of (2,2’-azino-bis (3-ethylbenzothiazoline-6-solfonic acid (ABTS) and ferric reducing/antioxidant power (FRAP) methods, and by Chludil and coworkers [24], who studied the influence of soil quality on *C. album* antioxidant potential. As regards *S. officinale*, the sample from Calabria here investigated showed a better biological activity compared to literature [25].

### 2.3. Anti-Inflammatory and Anti-Arthritic Potential

The anti-inflammatory potential of *C. album* and *S. officinale* extracts was first investigated through the assessment of their ability to inhibit the LPS-induced production of NO in the murine macrophage RAW 264.7 cell line. Cells were cultured with different concentrations of investigated samples in the presence of LPS (final concentration 1 µg/mL). The presence of nitrite, a stable oxidized product of NO, was verified in cell culture medium by means of the Griess reagent 24 h later.

Then, the potential role of *C. album* and *S. officinale* extracts in the treatment of arthritic disorders was assessed in vitro through the evaluation of their capacity to protect bovine serum albumin from heat denaturation. The denaturation of tissue proteins is a major cause of arthritic diseases. Thus, agents able to prevent protein denaturation could be useful for the development of new anti-inflammatory drugs [26]. 

**Figure 4:**
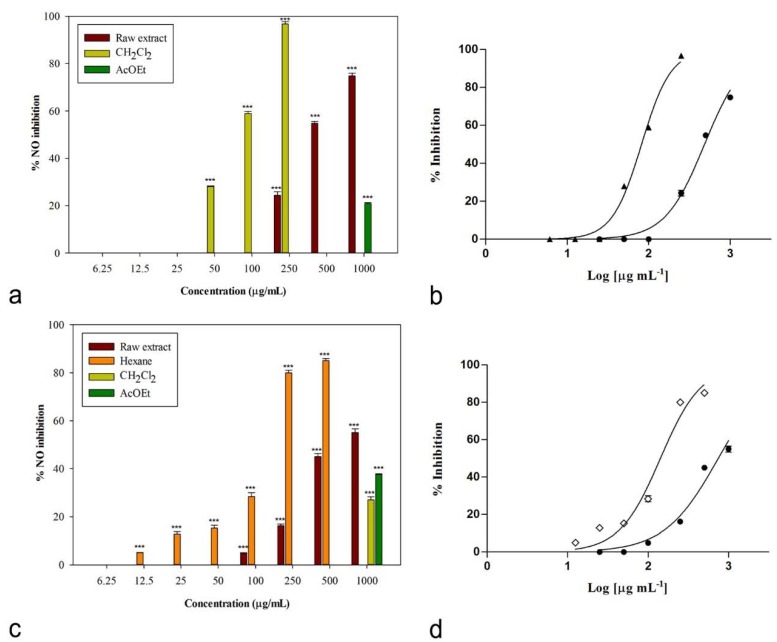
(**a**) Nitric oxide production inhibition induced by *Chenopodium album* L. (**b**) Non-linear regression analyses: ● raw extract, ▲ CH_2_Cl_2_ fraction. (**c**) Nitric oxide production inhibition induced by *Sisymbrium officinale* (L.) Scop. and (**d**) non-linear regression analyses: ● raw extract, ◊ *n*-hexane fraction. Data were expressed as means ± S.E.M. (n = 4). *** *P* < 0.001 compared to control (Dunnett’s test).

The observed nitric oxide production inhibition is reported in Figure 4. Cells were treated with different concentrations of raw extracts and fractions, ranging from 25 to 1000 μg/mL, with the only exception of the dichloromethane fraction of *C. album* (6.25–250 μg/mL) and the *n*-hexane fraction of *S. officinale* (12.5–500 μg/mL), as higher concentrations caused cytotoxic effects on the used macrophage cell line. The absence of cytotoxic effects on RAW 264.7 macrophages was verified for all the samples by means of the 3-(4,5-dimethylthiazol-2-yl)-2,5-diphenyltetrazolium bromide (MTT) assay. No toxic effects were detected for the other samples. 

Both raw extracts were effective in inhibiting nitric oxide production, with IC_50_ values equal to 483.2 ± 6.4 and 734.4 ± 21.2 μg/mL for *C. album* and *S. officinale*, respectively (Table 5). A good biological activity was observed for the *n*-hexane fraction of *S. officinale*, with an IC_50_ value of 142.0 ± 5.5 μg/mL. An excellent inhibitory activity was induced by the dichloromethane fraction of *C. album*, for which an IC_50_ value equal to 81.7 ± 0.9 μg/mL was calculated (Table 5). This significant result is interesting if compared to both positive controls (P < 0.05, Bonferroni post-hoc test), indomethacin (IC_50_ = 58.0 ± 0.9 μg/mL) and L-NAME (IC_50_ = 45.9 ± 0.5 μg/mL). This fraction significantly reduced LPS-induced synthesis of NO in a concentration-dependent manner (Figure 4a), inducing 96.76 ± 0.94% inhibition of NO production at the concentration of 250 μg/mL (P < 0.001, Dunnett’s multiple comparison test). Additionally, at 100 μg/mL the inhibition percentage was significant compared to control (*P* < 0.001).

**Figure 5:**
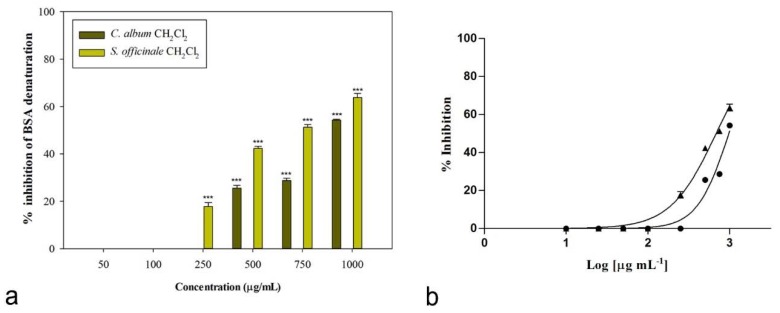
(**a**) Concentration-dependent anti-denaturation effects on heat-treated BSA induced by the dichloromethane fractions of investigated plant extracts. (**b**) Non-linear regression analyses: ● *C. album* CH_2_Cl_2_ fraction, ▲ *S. officinale* CH_2_Cl_2_ fraction.

Interestingly, the same fractions showed also a concentration-dependent anti-denaturation effect on heat-treated bovine serum albumin (IC_50_ = 975.6 ± 5.5 μg/mL, Table 5), even if, in this case, the highest activity was observed for the dichloromethane fraction of *S. officinale* extract, with an IC_50_ value of 680.9 ± 13.2 μg/mL. At the concentration of 1000 μg/mL, this fraction was able to induce 63.74% ± 1.77% inhibition of protein denaturation (Figure 5). At lower concentrations (750 and 500 μg/mL) inhibition percentages were 51.28% ± 1.09% and 42.36% ± 0.83%, respectively. The same fraction was still significantly effective at 250 μg/mL (17.82% ± 1.67%, *P* < 0.001, Dunnett’s multiple comparison test). 

Usman and coworkers investigated the anti-inflammatory potential of the essential oil of *C. album* leaves from Nigeria, reporting a significant reduction in 12-O-tetradecanoylphorbol-13-acetate (TPA)-induced ear edema in mice [27]. As regards *S. officinale*, a study conducted by Calcinoni demonstrated that this plant was able to reduce perceived disability in patients claiming vocal tract discomfort [5].

Our work adds interesting information about the anti-inflammatory potential of investigated species, demonstrating their effectiveness in inhibiting the production of the pro-inflammatory mediator nitric oxide. Moreover, to the best of our knowledge, this is the first report concerning the in vitro anti-denaturation effects on bovine serum albumin for these species.

## 3. Materials and Methods 

### 3.1. Chemicals

Bovine serum albumin, diclofenac sodium, potassium chloride, disodium hydrogen phosphate, sodium chloride, potassium dihydrogen phosphate, DMEM, FBS, PBS, MTT, L-NAME, L-glutamine, penicillin/streptomycin, trypan blue, Griess reagent, indomethacin, Folin-Ciocalteu reagent, aluminum chloride, ascorbic acid, DPPH, β-carotene, linoleic acid, propyl gallate, Tween 20, EDTA, sucrose, HEPES, trichloroacetic acid (TCA), butylated hydroxytoluene (BHT), hydrochloric acid, tert-butyl hydroperoxide, 2-thiobarbituric acid (TBA), and reference compounds utilized in HPTLC analyses were purchased from Sigma-Aldrich S.p.A. (Italy). The RAW 264.7 cell line was obtained from American Type Culture Collection (ATCC) no. TIB-71, UK. Normal phase glass plates were obtained from Merck (Germany). All the solvents used were reagent grade and were purchased from VWR International s.r.l. (Italy). 

### 3.2. Extraction Procedure

The aerial parts from wild plant species were collected in Southern Italy (Calabria) in July 2015 during the flowering stage (leg. F. Conforti, det. F. Conforti). Voucher specimens are deposited in the Herbarium CLU of our University (Table 1). Dried samples were extracted with methanol through maceration at room temperature (plant to solvent ratio 1:10 g/mL, 48 h × 3 times). Obtained solutions were then filtered and dried. A fraction of each raw extract (suspended in methanol:water, 9:1) was then extracted with n-hexane. The residue was then suspended in distilled water and extracted successively with dichloromethane and ethyl acetate. Samples were preserved at −20 °C.

### 3.3. Gas Chromatography-Mass Spectrometry (GC-MS) Analyses

The chemical composition of the *n*-hexane and dichloromethane fractions of the two raw extracts was assessed using a Hewlett-Packard 6890 gas chromatograph with an SE-30 capillary column 100% dimethylpolysiloxane (30 m length, 0.25 mm in diameter, 0.25 µm film thickness) coupled to a mass spectrometer Hewlett Packard 5973. Helium was used as carrier gas and analyses were run using a programmed temperature from 60 to 280 °C (rate 16 °C/min) with helium as carrier gas (linear velocity, 0.00167 cm/sec), as previously described [28]. The comparison of spectra with those of the Wiley 138 mass spectral library allowed the identification of compounds.

### 3.4. Total Phenolic Content and Flavonoid Content

The Folin Ciocalteau reagent was used to determine the total phenolic content of the two raw extracts as previously reported [29]. Briefly, 200 μL of each sample (2 mg/mL in acetone/MeOH/H_2_O/formic acid, 40:40:20:0.1) were added to 1 mL of Folin–Ciocalteau reagent and 1 mL of 7.5% w/v sodium carbonate. The absorbance was measured after two hours at 726 nm. 

Total flavonoid content was evaluated using a colorimetric method as earlier described [30]. The absorbance of a mixture consisting of sample (2 mg/mL in 80% EtOH) and 2% AlCl_3_ (1 mL) was measured at 430 nm after 15 min of incubation. Results were calculated from calibration curves based on the standards chlorogenic acid (for phenolics determination) or quercetin (flavonoids analysis), and were expressed as mg of standard equivalent per g of dry plant.

### 3.5. High-Performance Thin Layer Chromatography (HPTLC) Analyses

The constituents of the polar fractions from *C. album* and *S. officinale* were identified by means of high-performance thin layer chromatography (HPTLC) by means of the CAMAG semi-automated HPTLC system including a Linomat 5 sample applicator connected to a TLC Visualizer and controlled with wincats planar chromatography software. 

Samples and reference compounds were dissolved in methanol to a final concentration of 50 and 3 mg/mL, respectively, and spray-applied with a micro-syringe on 20 × 10 cm silica gel glass plates (silica 2–10 µm; 2 μm thickness). Operating conditions were the same as previously described [31]. Plates were developed using a mixture of ethyl acetate/dichloromethane/acetic acid/formic acid/water (100:25:10:10:11, v/v/v/v/v). For post-chromatographic derivatization, plates were dipped in freshly prepared NPR reagent and anisaldehyde reagent, and heated at 100 °C for 5 min. The first reagent was prepared by dissolving diphenylborinic acid aminoethyl ester (1 g) in AcOEt (200 mL). The second one by mixing *p*-anisaldehyde (1.5 mL), sulfuric acid (2.5 mL), AcOH (1 mL), and ethanol (37 mL). The plates were examined before and after derivatization under UV light (254 or 366 nm) and white light. Samples were analyzed by co-chromatography with the reference compounds rutin, chlorogenic acid, cinnamic acid, caffeic acid, sinapic acid, *p*-coumaric acid, naringenin, quercetin, kaempferol, luteolin, ferulic acid, catechin, and naringin. 

### 3.6. Free Radical Scavenging Activity (FRSA) Assay

The DPPH method was used to evaluate the radical scavenging activity of extracts and their fractions. The radical 2,2-diphenyl-1-picrylhidrazyl (DPPH, 0.1 mM in MEOH, 0.8 mL) was mixed with samples (0.2 mL) at concentrations ranging from 5 to 1000 µg/mL (or to ascorbic acid in the positive control group). Absorbance was measured at 517 nm 30 min later [32]. 

### 3.7. β-Carotene Bleaching Test

The antioxidant activity was evaluated using the β-carotene bleaching test. An emulsion was prepared by adding a β-carotene solution (1 mL, 0.5 mg/mL in CHCl_3_) to linoleic acid (0.02 mL) and 100% Tween 20 (0.2 mL), removing chloroform and adding distilled water (100 mL). Five milliliters of obtained emulsion was mixed with 0.2 mL of different samples solutions (0.25–100 μg/mL), or to propyl gallate in the positive control group, and incubated at 45 °C. Absorbance was measured at 470 nm at different times (initial time, 30 min, and 60 min). The prevention of β-carotene bleaching indicated the antioxidant activity of samples [33]. 

### 3.8. Microsomal Suspensions

The microsomal suspension was prepared from liver of Wistar rats homogenizing the tissue in a PotterElvehjem with a solution, pH 7.5, containing 0.25 M sucrose, 5 mM HEPES, and 0.5 mM EDTA [20,34]. Microsomes were isolated by the nuclear fraction at 8000 g for 10 min and subsequently by mitochondrial fraction at 18,000 g for 10 min. The microsomal fraction was sedimented at 105,000 g for 60 min, washed in 0.15 M KCl, and collected again at 105,000 g for 30 min [35]. The microsomes were suspended in 0.1 M potassium phosphate buffer, pH 7.5, and stored at −80 °C. The protein concentration was determined by the Bio-Rad method [36].

#### 3.8.1. Addition of Extracts to Microsomes 

Aliquots of extracts were added to the microsomes to give concentrations in the range 0.1–1 mg/mL. Control samples were treated with a water amount equal to those present in extracts-treated microsomes. These were suspended and then were incubated at 37 °C in the dark in the presence of t-BOOH.

#### 3.8.2. Malondialdehyde Formation

Aliquots of 1 mL of microsomal suspension were mixed with a solution composed of TCA, TBA, and BHT in 95% ethanol, and then incubated in a 40 °C bath for 1 h. Subsequently, the TBA-MDA complex was extracted with 3 mL of isobutyl alcohol and malondialdehyde (MDA) was measured at 535 nm.

### 3.9. Nitric Oxide Production Inhibition 

The ability of plant extracts and their fractions to inhibit NO production was verified in vitro on RAW 264.7 cells stimulated with LPS. Dulbecco’s modified Eagle’s medium (DMEM) was used as growth medium. It was supplemented with L-glutamine, fetal bovine serum, and a solution of penicillin and streptomycin (1%, 10% and 1%, respectively). Cells were cultured at 37 °C under 5% CO_2_. For the experiments, cells were removed from flask by scraping and then seeded onto microplates (96 wells, 100,000 cells/well). After 24 h, medium was removed and fresh DMEM containing samples at different concentrations (6–1000 μg/mL in DMSO, final ratio of DMSO to medium 0.5% v/v) and 1 μg/mL LPS was added. After 24 h of incubation, the Griess reagent was used to evaluate the presence of nitrite, a stable end product of nitric oxide oxidation, in cell culture media. A total of 100 μL of Griess reagent was added to 100 μL of cell culture supernatant. Absorbance was measured at 550 nm. Indomethacin and the NO synthase inhibitor L-NAME were used as positive controls [37].

The absence of cytotoxic effects on RAW 264.7 macrophages was verified by means of the MTT assay [38]. At the end of the experiments, MTT (0.5%, 100 μL/well) was added to the wells. Four hours later, DMSO (100 μL/well) was also added and absorbance was measured at 550 nm.

### 3.10. Anti-Arthritic Potential

The anti-arthritic potential of *C. album* and *S. officinale* extracts and fractions was evaluated by means of the in vitro protein denaturation assay reported by Palit and colleagues [39] with some modifications.

To realize the experiment, 2.40 mL of a 3.5% bovine serum albumin water solution (BSA) were mixed with 0.10 mL of samples (concentrations ranging from 1000 to 50 µg/ml in DMSO). Diclofenac sodium (250 μg/mL) was used as positive control. pH was adjusted at 6.3 using 1N HCl and samples were then incubated at 37 °C for 20 min and then heated at 71 °C for 1 min. After cooling, phosphate buffered saline (pH 6.3, 2.5 mL) was added to each sample. Buffer was prepared by dissolving 8 g of NaCl, 0.2 g of KCl, 1.44 g of Na_2_HPO_4_, and 0.24 g of KH_2_PO_4_ in 800 mL of distilled water; pH was adjusted at 6.3 using 1N HCl and the final volume was brought to 1000 mL with distilled water. Diclofenac sodium (250 μg/mL) was used as positive control.

The turbidity of obtained solutions was measured spectrophotometrically at 660 nm. Product control groups were prepared without bovine serum albumin and the percentage of protein denaturation inhibition was calculated as shown in the following equation:

Inhibition % = [1 − (Abs test solution − Abs product control) / Abs untreated control] × 100.(1)

### 3.11. Statistical Analysis

Experiments were run in triplicate, except for tests involving cell cultures, for which four replicates were performed. Data were expressed as means ± S.E.M. Normality of data and homogeneity of variances were assessed using D’Agostino–Pearson’s K2 test and Levene’s test, respectively. Nonlinear regression analyses were performed using Graph-Pad Prism Software (San Diego, CA, USA) in order to deduce the IC_50_ parameters. One-way ANOVA was carried out to test the statistical differences between treated groups and the control (Dunnett’s multiple comparison test) and among treated groups means (Bonferroni post-hoc test, *P* ≤ 0.05, SigmaStat Software, SanRafael, CA, USA). 

## 4. Conclusions

Botanicals may potentially play an important role in the treatment of anti-inflammatory diseases, with the aim to avoid common undesired side effects of the main synthetic drugs commonly utilized [40,41]. Herbal remedies and dietary plants used in traditional medicine could be a promising source of new effective drugs [42,43]. 

In this context, the results of this work proved in vitro the potential effectiveness of *C. album* and *S. officinale*, two traditionally used herbal drugs in the treatment of inflammatory disorders. Both species demonstrated to inhibit the production of the pro-inflammatory mediator nitric oxide in LPS-stimulated murine macrophages in a concentration-dependent manner, the dichloromethane fraction of *C. album* being the most active sample (IC_50_ = 81.7 ± 0.9 μg/mL). Moreover, some samples demonstrated an interesting in vitro anti-arthritic effect in experimental studies, showing significant protein anti-denaturation effects, verified on heat-treated bovine serum albumin. Additionally, in this case, *C. album* dichloromethane fraction was effective, even if to a lesser extent compared to the same fraction of the second species, *S. officinale*, with IC_50_ values, respectively, equal to 975.6 ± 5.5 and 680.9 ± 13.2 μg/mL.

Furthermore, both plant species demonstrated in vitro antioxidant properties, verified by means of the DPPH and β-carotene bleaching methods. The activity of extracts against lipid peroxidation was also verified in microsomal suspension from rat liver. All extracts were effective antioxidants against tert-BOOH-induced lipid peroxidation, showing a preservation of their activity up to one hour.

In conclusion, the results reported in this study seem to support the traditional use of *C. album* and *S. officinale* in the treatment of inflammatory conditions.

## Figures and Tables

**Table 1:** Investigated plants: Extraction yields and total phenolic and flavonoid contents.

Botanical Name	Family	Voucher Number	Yield (%)	TP ^1^	TF ^2^
*Chenopodium album* L.	Amaranthaceae	26247	23.2	12.8 ± 1.6	0.77 ± 0.01
*Sisymbrium officinale* (L.) Scop.	Brassicaceae	26236	10.6	8.1 ± 0.1	0.50 ± 0.01

^1^ Total phenolic content. ^2^ Total flavonoid content. Data are expressed as mean ± SE (n = 3). Results were expressed as mg of chlorogenic acid or quercetin equivalent *per* g of dry plant material, respectively.

**Table 2:** Phytochemical profile of *n*-hexane fractions of *Chenopodium album* L. and *Sisymbrium officinale* (L.) Scop.

Compound ^1^	Rt ^2^	RAP ^3^	
		*C. album* L.	*S. officinale* (L.) Scop.
**Fatty Acids**			
Caprylic acid	10.106	Tr ^4^	Tr
Pelargonic acid	12.00	-	0.1
Lauric acid	15.044	Tr	0.1
Myristic acid	16.799	0.7	0.3
4,8,12-Trimethyltridecanoic acid	16.930	-	0.1
Pentadecanoic acid	17.616	Tr	0.2
Tetradecanoic acid, 5,9,13-trimethyl-	17.708	Tr	-
Palmitelaidic acid	18.096	Tr	-
Palmitic acid	18.159	15.7	10.0
Oleic acid	18.531	Tr	-
Margaric acid	18.902	0.3	-
Isooleic acid	19.456	0.8	-
Stearic acid	19.634	1.3	1.0
Arachidic acid	20.988	0.8	1.0
Linoleic acid	21.051	-	0.5
Behenic acid	22.274	0.9	0.8
Tricosylic acid	22.966	Tr	0.4
Lignoceric acid	23.731	-	0.6
Pentacosylic acid	24.696	-	0.3
Cerotic acid	25.829	-	0.9
Montanic acid	28.847	-	tr
**Terpenes**			
Dihydroactinidiolide	14.987	0.9	0.4
Neophytadiene	17.473	0.7	-
**Phytosterols**			
β-Sitosterol	33.882	3.2	-

^1^ Compounds listed in order of elution from SE30 MS column. ^2^ Retention time (as min). ^3^ Relative area percentage (peak area relative to total peak area in total ion current (TIC) %). ^4^ Tr: Traces percentages < 0.1%.

**Table 3:** Chemical composition of dichloromethane fractions of *Chenopodium album* L. and *Sisymbrium officinale* (L.) Scop. methanolic extracts.

Compound ^1^	Rt ^2^	RAP ^3^	
		*C. album* L.	*S. officinale* (L.) Scop.
Phenol	7.677	-	Tr ^4^
Benzoic acid	10.758	1.0	-
Methylethylmaleimide	11.575	1.4	0.3
2,4-Di-tert-butylphenol	14.621	-	Tr
Loliolide	17.073	1.7	-
Coniferyl alcohol	17.250	-	1.2
Ferulic acid	17.565	-	0.7

^1^ Compounds listed in order of elution from SE30 MS column. ^2^ Retention time (as min). ^3^ Relative area percentage (peak area relative to total TIC peak area %). ^4^ Tr: Traces percentages < 0.1%.

**Table 4:** In vitro antioxidant activity of plants extract and fractions.

Species	Sample	IC_50_ (µg/mL)		
		DPPH Test	β-carotene Bleaching Test	
			30 min	60 min
*C. album* L.	Raw extract	172.70 ± 2.18 ^d^	60.51 ± 2.34 ^e^	˃100
	*n*-Hexane	˃1000	˃100	˃100
	CH_2_Cl_2_	435.60 ± 12.97 ^f^	˃100	˃100
	EtOAc	140.40 ± 4.36 ^c^	12.07 ± 0.04 ^b^	38.03 ± 1.88 ^d^
	H_2_O	˃1000	˃100	˃100
*S. officinale* (L.) Scop.	Raw extract	143.00 ± 2.61 ^c^	2.61 ± 0.06 ^a,b^	8.53 ± 0.27 ^b^
	*n*-Hexane	˃1000	˃100	˃100
	CH_2_Cl_2_	˃1000	61.02 ± 2.31 ^e^	˃100
	EtOAc	60.11 ± 1.79 ^b^	12.62 ± 0.75 ^b^	30.49 ± 1.17 ^c^
	H_2_O	262.9 ± 0.93 ^e^	˃100	˃100
Ascorbic acid ^1^		2.00 ± 0.01 ^a^	-	-
Propyl gallate ^1^		-	1.00 ± 0.02 ^a^	1.00 ± 0.02 ^a^

Data are expressed as mean ± SEM (n = 3). Different letters along column (DPPH test) or between columns (β-carotene bleaching test) indicate statistically significant differences at *P* < 0.05 (Bonferroni post-hoc test). ^1^ Positive controls.

**Table 5:** In vitro inhibitory activity on NO production and anti-arthritic potential.

Species	Sample	IC_50_ (µg/mL)	
		NO Inhibition	BSA Denaturation Inhibition
*C. album* L.	Raw extract	483.2 ± 6.4 ^c^	n.a.
	*n*-Hexane	n.a.	n.a.
	CH_2_Cl_2_	81.7 ± 0.9 ^a^	975.6 ± 5.5 ^c^
	EtOAc	n.a.	n.a.
	H_2_O	n.a.	n.a.
*S. officinale* (L.) Scop.	Raw extract	734.4 ± 21.2 ^d^	n.a.
	*n*-Hexane	142.0 ± 5.5 ^b^	n.a.
	CH_2_Cl_2_	n.a.	680.9 ± 13.2 ^b^
	EtOAc	n.a.	n.a.
	H_2_O	n.a.	n.a.
Indomethacin ^1^		58.0 ± 0.9 ^a^	-
L-NAME ^1^		45.9 ± 0.5 ^a^	-
Diclofenac ^1^		-	15.73 ± 0.2 ^a^

Data are expressed as mean ± SEM (n = 4, NO inhibition; n= 3, Bovine serum albumin (BSA) denaturation). Different letters along columns indicate statistically significant differences at P < 0.05 (Bonferroni post-hoc test). n.a. = not active. ^1^ Positive controls.
